# Recognition in Ants: Social Origin Matters

**DOI:** 10.1371/journal.pone.0019347

**Published:** 2011-05-04

**Authors:** Joël Meunier, Olivier Delémont, Christophe Lucas

**Affiliations:** 1 Department of Ecology and Evolution, University of Lausanne, Lausanne, Switzerland; 2 Zoological Institute, Evolutionary Biology, University of Basel, Basel, Switzerland; 3 Institut de Police Scientifique, Ecole des Sciences Criminelles, University of Lausanne, Lausanne, Switzerland; 4 Institut de Recherche sur la Biologie de l'Insecte (UMR 6035), CNRS, University of Tours, Tours, France; Texas A&M University, United States of America

## Abstract

The ability of group members to discriminate against foreigners is a keystone in the evolution of sociality. In social insects, colony social structure (number of queens) is generally thought to influence abilities of resident workers to discriminate between nestmates and non-nestmates. However, whether social origin of introduced individuals has an effect on their acceptance in conspecific colonies remains poorly explored. Using egg-acceptance bioassays, we tested the influence of social origin of queen-laid eggs on their acceptance by foreign workers in the ant *Formica selysi*. We showed that workers from both single- and multiple-queen colonies discriminated against foreign eggs from single-queen colonies, whereas they surprisingly accepted foreign eggs from multiple-queen colonies. Chemical analyses then demonstrated that social origins of eggs and workers could be discriminated on the basis of their chemical profiles, a signal generally involved in nestmate discrimination. These findings provide the first evidence in social insects that social origins of eggs interfere with nestmate discrimination and are encoded by chemical signatures.

## Introduction

The evolution of sociality requires recognition mechanisms allowing group members to direct cooperative or aggressive behaviours towards the correct individuals. In particular, the ability to discriminate against non-group members is important as it may help to prevent colony invasion, to avoid exploitation of group resources by outsiders and to limit erosion of relatedness between group members [1], [2]. Identifying which factors influence nestmate discrimination is therefore fundamental to gain a better understanding of social evolution.

In social insects, nestmate recognition is generally mediated by the blend of chemical compounds (CC) present on the cuticle of individuals [3]. These CC are mainly hydrocarbons, which can have genetic bases and also be acquired from the environment [4]. Repeated grooming, trophallaxis and body contacts between individuals regularly homogenize chemical profiles among colony members. This blend of chemicals produce a colony-profile, which compared to intruders' odours, allows resident workers to assess colony membership and engage correct behavioural responses [3], [5]–[7]. Colony social structure is generally thought to influence abilities of resident workers to discriminate between nestmates and non-nestmates. In particular, the presence of multiple resident queens is predicted to broaden the mix of genetically determined chemical cues composing the colony-profile, which in turn is expected to increase discrimination errors between nestmate and non-nestmates individuals, and therefore to inhibit aggression against conspecific foreigners [8], but see [9].

Odours of intruders may also influence nestmate discrimination through distinct pathways. One way is to present a relatively small global-amount of CC, so that resident workers are not able to compare intruders' odours to colony-profile [10]. Recent studies in wasps and ants support this hypothesis, showing that lure presenting relatively small global amount of cuticular hydrocarbons elicit fewer aggressions from foreign workers [11]–[13]. The other possibility is to present a specific blend of CC that interferes with nestmate discrimination. For instance in the ant *Camponotus floridanus*, queens and queen-laid eggs presenting CC associated with high fertility rates elicit fewer aggression from foreign conspecific workers [14], [15].

Signals associated with the social origin of intruders are also known to influence discrimination against foreign queens and workers. In the fire ant *Solenopsis invicta*, workers from multiple-queen ( =  polygyne) colonies tolerated foreign conspecific queens only if these queens came from polygyne colonies [16]. In the ant *Messor barbatus*, workers from single-queen ( =  monogyne) colonies accepted more foreign conspecific workers from polygyne than monogyne colonies [17]. Finally in the ant *Formica selysi*, resident workers were less aggressive towards foreign workers originating from colonies with similar than alternative social forms [18]. Chemical analyses suggested that discrimination between intruders from alternative social origins could be based on chemical profiles, as *S. invicta* queens and *M. barbatus* workers present subtle differences of chemical profiles when originating from monogyne and polygyne colonies [19], [20]. Whether chemical profiles of *F. selysi* workers reflect their social origin remained however untested so far.

Contrary to adult intruders, the influence of social origin of foreign eggs (we always refer to queen-laid eggs in this manuscript) on their elimination by resident workers has never been explored in social insects. The elimination of foreign eggs can be important to maintain colony integrity, as subsequent brood from infiltrated queens may dilute relatedness among nestmates, monopolise colony resources, parasitize host colonies and hence can decrease inclusive fitness of resident workers and induce colony collapse [2], [21]. In *F. selysi*, recent works suggest that social origin of foreign eggs could interfere with their elimination rate. In particular, monogyne workers rejected eggs from foreign monogyne colonies, while polygyne workers accepted eggs from foreign polygyne colonies [22]. These results called for further studies disentangling whether social origin of introduced eggs or recipient workers influenced the acceptance of foreign eggs, and testing potential association between chemical profiles and social origin of eggs.

Our present study was conducted in the ant *F. selysi* (i) to test the influence of social origin of eggs on their acceptance by foreign workers, and (ii) to investigate the association between chemical profiles and social origins of workers and eggs. Using egg-acceptance bioassays, we first investigated whether monogyne and polygyne workers discriminated between eggs from their own colony, foreign monogyne colony and foreign polygyne colony. Then, we determined whether social origin of eggs and workers could be discriminated on the basis of their chemical profiles by testing qualitative and quantitative differences between chemical profiles of individuals (i.e. eggs and workers) sampled in 23 monogyne and 23 polygyne field colonies.

## Materials and Methods

### Model species

The study population of *F. selysi* is located between Sierre and Susten along the river Rhône in central Valais, Switzerland (7°36′30″E, 46°18′30″N, altitude 565 m). In this population, monogyne and polygyne colonies live in close proximity with no sign of genetic differentiation or mating incompatibilities between social forms [23], [24]. The social structure (monogyne or polygyne) of each colony involved in this study had been previously determined by genotyping eight to 100 workers per colony at nine microsatellite markers (method described in [23]). Repeated sampling and genotyping of individuals in the same colonies over several years confirmed that the colonies have stable social structures with very low rates of queen turnover [23], [25], [26].

### Egg acceptance bioassays

We estimated the survival rate of eggs introduced into 93 recipient groups of workers using the set up described in Meunier *et al.* [22]. Workers and eggs were sampled from 25 monogyne and 18 polygyne field colonies during the first week of May 2008. Each recipient group was composed of 100 nestmate workers placed in a fluon-lined plastic box (15×15×15 cm) with access to standard ant food *ad libitum* (food composition in [27]). The day of field sampling, each recipient group of workers was set up and received a set of 30 eggs (8 out of the 93 tested groups of workers received (mean ± SD) 17.5±4.2 eggs due to the small quantity of eggs found) from either (i) nestmate colonies, (ii) foreign monogyne colonies or (iii) foreign polygyne colonies. Prior to introduction, eggs were placed in small plastic trays (3×3 cm) and observed under a stereomicroscope to check that they were not damaged. Twenty-four hours after introduction, the number of undamaged eggs in each recipient group of workers was counted. Egg survival rate was the ratio between the number of undamaged eggs counted after 24 hours and the total number of introduced eggs.

### Chemical analyses

Extractions of the CC were made on groups of 10 workers and 30 eggs. Workers were randomly collected under large flat stones covering the 23 monogyne and 23 polygyne colonies used for chemical analyses [28], and immediately frozen on dry ice. Eggs were collected at the same place than workers and individually observed to exclude damaged ones from extractions. On the field, groups of frozen workers and sampled eggs were placed into glass vials (2 ml, Sigma-Aldrich, Buchs, Switzerland) filled with 500 µl of hexane (Sigma 52765, Buchs, Germany). Five minutes later, workers and eggs were removed from hexane, and their respective vials were sealed and stored at −20°C. Both eggs and workers were collected and chemical profiles extracted on the 7^th^ and 15^th^ of April 2009.

Chemical analyses were made on the hexane extracts described above, which were previously evaporated and reconstituted in 100 µl of hexane with 10 ng/µl of eicosane (*n*C20; not present in *F. selysi*) as an injection internal standard. A 2 µl sample of the extract was injected on a Agilent 7890 gas chromatograph fitted with a HP-5MS fused silica capillary column (0.25 mm×30 m, 0.25 µm film thickness; Agilent, Morges, Suisse) linked to a mass analyzer (Agilent 5975 mass spectrometer). The injector was used in splitless mode with a splitless time of 2 min. Injector temperature was held constant at 250°C. An oven program that began at 70°C (1 min) and was ramped at 20°C/min to 140°C, then 3°C/min to 230°C, 2°C/min to 260°C and 3°C/min to 300°C (10 min). Carrier gas was Helium at a flow rate of 1 ml/min. Electron impact positive ions at 70 eV were recorded in the scanning mode (mass range scanned 40–550 amu). The mass spectra were interpreted by fragmentation analysis and comparison to previous publications [29]–[32]. Retention indices based on a series of *n*-alkane standards (C24–C30, Grace GR-628008) were compared to published data. MSD Chemstation Agilent Technologies software was used to calculate the retention time and total area of each peak for subsequent analysis.

### Statistical analyses

The survival of eggs was tested using mixed-effect models (GLMMs) within groups of monogyne and polygyne workers. In these analyses, the origin of eggs (nestmate, foreign from monogyne colonies or foreign from polygyne colonies) was used as a fixed factor and the arcsine-transformed proportion of eggs still alive after 24 hours (egg survival rate) entered as response variable. The normality of residuals were tested using Shapiro Wilcoxon tests (all *p*>0.05), and pairwise comparisons between egg origins tested using post-hoc Tukey HSD tests. Because eggs or workers originating from the same field colony were sometimes used twice in the analyses (albeit once per egg origin), we included the colony of origin of eggs and workers as random factors in all the analyses.

Whether social origin of eggs and workers could be discriminated on the basis of their chemical profiles was tested using two linear discriminant analyses (DA). The significance of each DA was evaluated (i) by testing difference between groups using Wilks' Lamba tests and (ii) through the percentage of correct assignment of eggs or workers to their social origin, which was given by statistical models and cross-validations (Leave-one-out method). The structure coefficients (i.e. correlations between discriminating variables and discriminant functions) were used to assess the importance of each peak in discriminating social origin of eggs and workers. According to Mardia's criterion [33], coefficient of correlations above 0.7 times the largest coefficient in a discriminant function were considered to have contributed significantly. Coefficients of correlations were obtained from Spearman rank correlation tests.

Because a sample size of at least three times the number of variable is recommended for multivariate analyses [34], DAs were done on 15 out of all the extracted peaks (table 1). We selected the 15 peaks with the highest variations between social forms rather than the ones with the highest relative amount, because no clear evidence exists about positive associations between relative amount of CC and importance of information [35]. Variation between social forms was estimated using Mann-Whitney *U-*tests. To avoid limitations inherent to analyses of compositional data, the area of each peak was transformed according to Aitchison formula [36] prior to DAs. In this formula, 
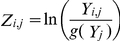
, *Z_i,j_* is the transformed area of peak *i* for colony *j*, *Y* is the area of the peak *i* for colony *j*; and *g(Y_j_)* is the geometric mean of the areas of all peaks of the colony *j*. To apply this formula in profiles containing undetected components, the constant 1750 ( =  one-tenth of the smallest area measured) was added to all peak areas [35]. DA on the absolute quantity of each peak provided comparable results.

**Table 1:** Mean relative amount (%) of the chemical compounds used in discriminant function analyses.

				Workers					Eggs				
Peaks	RT	Data set		Mo	Po	*P*-value	*r*_s_		Mo	Po	*P*-value	*r*_s_	
1	4.56	W		**0.261**	**0.056**	**0.0004**	*−0.52*		0.076	0.053	0.0106		
2	6.42	W		0.068	0.043	0.0255	*−0.33*		*−*	*−*	*−*		
4	8.23	W		0.119	0.078	0.0381	*−0.32*		*−*	*−*	*−*		
10	15.17		E	0.035	0.045	0.0448			0.107	0.088	0.0009	*−0.54*	*
15	19.48		E	0.104	0.084	0.3712			0.086	0.073	0.0009	*−0.55*	*
22	25.87		E	0.010	0.008	0.3596			0.038	0.027	0.0016	*−0.52*	*
27	28.03		E	0.050	0.055	0.5276			**0.318**	**0.486**	**0.0003**	*0.58*	*
29	30.05	W	E	1.049	1.572	0.0146	*0.40*		**1.473**	**2.180**	**0.0003**	*0.58*	*
30	30.22		E	0.051	0.061	0.6013			**0.084**	**0.141**	**<0.0001**	*0.70*	*
31	30.82		E	1.808	1.708	0.4199			8.884	12.572	0.0015	*0.49*	*
34	30.77		E	0.012	0.009	0.0826			0.036	0.023	0.0009	*−0.56*	*
35	31.74	W		**0.105**	**0.506**	**<0.0001**	*0.76*	*	0.858	1.035	0.0505		
36	31.92	W		0.059	0.095	0.0015	*0.46*		*−*	*−*	*−*		
37	32.11	W		0.062	0.093	0.0039	*0.39*		0.168	0.130	0.0546		
39	32.70	W	E	1.024	1.452	0.0121	*0.43*		**0.211**	**0.297**	**0.0002**	*0.57*	*
52	36.23	W		**9.286**	**5.241**	**<0.0001**	*−0.61*	*	29.213	24.053	0.0130		
56	36.99	W		0.298	0.539	0.0092	*0.40*		0.959	0.873	0.1064		
61	38.06		E	2.090	2.139	0.9307			**0.738**	**0.930**	**0.0006**	*0.55*	*
65	39.01	W	E	**0.776**	**1.276**	**<0.0001**	*0.78*	*	**0.285**	**0.451**	**<0.0001**	*0.69*	*
77	43.67	W	E	1.301	2.031	0.0080	*0.45*		0.838	1.028	0.0009	*0.49*	
79	44.68	W	E	**0.904**	**2.308**	**<0.0001**	*0.81*	*	0.755	0.992	0.0007	*0.53*	*
85	46.79		E	4.422	4.440	0.6013			4.320	5.161	0.0009	*0.53*	*
89	48.30	W		0.269	0.207	0.0240	*−0.29*		0.355	0.333	0.4485		
95	50.41	W		**0.305**	**0.779**	**<0.0001**	*0.72*	*	0.380	0.335	0.0018		
98	52.25		E	0.504	0.509	0.5713			**1.040**	**1.480**	**<0.0001**	*0.64*	*

Mean retention times (RT) are given in minutes. Discriminant analyses were computed on data set including groups of (*W*) workers or (*E*) eggs originating from (*Mo*) monogyne and (*Po*) polygyne colonies. Values in **bold** remained significant after Bonferroni correction (reported *P*-values are uncorrected). Correlations between the relative amount of each peak and the respective discriminant function are provided (*r_s_*). Asterisks (*) indicate chemical compounds that significantly contribute in discriminating social origins of workers or eggs [33]. ^(Peak 1)^ Nonanal; ^(Peak 2)^ *n*C13; ^(Peak 4)^ Tridecanol;^(Peak 10)^ Butyl dodecanoate; ^(Peak 15)^ *n*C19; ^(Peak 22)^ Nonadecanal; ^(Peak 27)^ *n*C22; ^(Peak 29)^ 9-C23∶1; ^(Peak 30)^ 7-C23∶1; ^(Peak 31)^ *n*C23; ^(Peak 34)^ Heneicosanal; ^(Peak 35)^ 11-,9-MeC23; ^(Peak 36)^ 7-MeC23; ^(Peak 37)^ 5-MeC23; ^(Peak 39)^ 3-MeC23 + Decyl dodecanoate; ^(Peak 52)^ *n*C25; ^(Peak 56)^ 13-,11-,9-MeC25; ^(Peak 61)^ 3-MeC25 + Decyl tetradecanoate + Dodecyl dodecanoate; ^(Peak 65)^ 3,9-+ 3,7-di-MeC25 + Decyl pentadecanoate + Undecyl tetradecanoate + Dodecyl tridecanoate; ^(Peak 77)^ 3-MeC27 + Dodecyl tetradecanoate + Decyl hextadecanoate; ^(Peak 79)^ x,y-diMeC28 (mainly) *+ n*C28; ^(Peak 85)^ 9-C29∶1 ; ^(Peak 89)^ 11-,9-MeC29; ^(Peak 95)^ 12-,10-,8-MeC30; ^(Peak 98)^ 9-C31∶1 (mainly) + 9,23-C31∶2.

Differences in the total amount of CC extracted from groups of monogyne and polygyne eggs or workers were investigated using *t*-tests. For each sampled colony, the total absolute quantity of extracted CC was calculated using the formula 
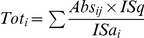
, where *Tot_i_* is the total absolute quantity of CC extracted from colony *i*, *Abs_ij_* is the GC-MS area of the peak *j* for colony *i, ISq* is the quantity of internal standard introduced in the sample (here 20 ng) and *ISa_i_* is the GC-MS area of the internal standard in the colony *i*.

## Results

### Egg discrimination

**Figure 1:**
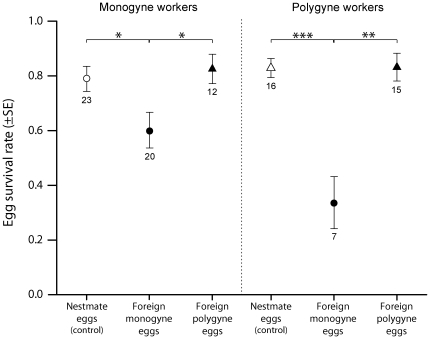
Survival rate of eggs introduced in groups of monogyne and polygyne workers. In both groups of workers, foreign monogyne eggs (*black circle*) had a significantly lower survival rate than both foreign polygyne eggs (*black triangle*) and nestmate eggs (*white circle* and *white triangle*), whereas there was no significant difference between the survival rates of foreign polygyne eggs and nestmate ones. The number of recipient group is indicated below the SE bars. * *p*<0.05, ** *p*<0.01, *** *p*<0.001.

The social origin of foreign eggs significantly influenced their survival rate in both groups of monogyne and polygyne workers (figure 1, GLMMs, monogyne workers: *F*_2,30.96_ = 5.19, *p* = 0.011; polygyne workers: *F*_2,17.94_ = 13.75, *p*<0.001). Overall, foreign eggs originating from monogyne colonies survived significantly less than nestmate eggs (Tukey HSD tests, monogyne workers: *p* = 0.026; polygyne workers: *p*<0.001) or foreign eggs from polygyne colonies (monogyne workers: *p* = 0.029; polygyne workers: *p*<0.001). By contrast, there was no significant difference between the survival rates of nestmate eggs and foreign polygyne eggs (monogyne workers: *p* = 0.868; polygyne workers: *p* = 0.892). A lower intrinsic viability of monogyne than polygyne eggs is unlikely to explain these results, as there was no significant difference between the survival rates of both types of eggs introduced with nestmate workers (figure 1, *white circle* and *white triangle*, *t*-test, *t* = 0.62, d.f. = 37, *p* = 0.54).

### Chemical profiles of eggs and workers

Chemical profiles of eggs and workers contained qualitative differences. Thirty-two peaks are exclusively present in workers and one is present in eggs only (table S1). Chemical profiles generally contained a majority of hydrocarbons like alkanes, methylalkanes, odd di-methylalkanes and linear unsaturated hydrocarbons (mono- and di-enes). Chain lenghts are even- or odd-numbered and range from 9 to 33 carbons atoms. Major peaks (more than 5% of total area) are composed of linear alkanes such as tricosane (*n*C23), pentacosane (*n*C25) and heptacosane (*n*C27) or 9-monoenes (9-C25∶1, 9-C27∶1, 9-C29∶1) (respectively peaks 31, 52, 72, 48, 70 and 85). Peaks 1 to 9 are very volatile compounds and are usually associated with the Dufour gland [29], [37]. They are present only in small quantities in workers and eggs from both social forms. A few other compounds rarely described in *F. selysi* were found in large quantities like aldehydes, alcohols and acid esters [29], [37]. Because of the presence of acids, peaks were generally more complex in workers than eggs, whatever the social form. Indeed, acids are present only in workers not in eggs (except in peak 10, 24 and 53).

### Social origins and chemical signatures

After Bonferonni correction, the relative amount of 7 out of 67 peaks extracted from eggs and 6 out of 98 peaks extracted from workers were significantly different between monogyne and polygyne colonies (tables 1 and S1). Those peaks were mainly composed of linear alkanes (peaks 15, 27, 31, 52), branched alkanes (peaks 35, 39, 61, 77, 95), mono alkenes (peaks 29, 30, 85, 98), aldehydes (peaks 1, 22 and 34) and dimethyl alkanes (peak 65, 79).

Discriminant analyses clearly separated workers and eggs according to their social origins. Discriminant scores from the two DAs were significantly larger in polygyne than monogyne groups of workers and eggs (workers: *Wilks' lambda* = 0.117, *F*_1,30_ = 15.05, *p*<0.0001; eggs: *Wilks' lambda* = 0.225, *F*_1,30_ = 6.90, *p*<0.0001). Moreover, 97.8% of workers and eggs were correctly assigned to their social origins by statistical models, and respectively 91.3% and 84.8% by cross-validation method. According to Mardia's criterion, five and 14 out of the 15 selected peaks significantly contributed in discriminating social origin of workers and eggs, respectively (table 1).

The total amount of extracted CC was significantly larger in polygyne than monogyne eggs (polygyne: 388.2±20.2 ng (mean ± SE); monogyne: 335.3±14.7 ng; *t*-test, *t* = 2.11, d.f.  = 44, *p* = 0.040), and significantly smaller in polygyne than monogyne workers (polygyne: 1836.7±94.2 ng; monogyne: 2092.2±79.0 ng; *t*-test, *t = *2.08, d.f. = 44, *p* = 0.044). Despite these differences, the social origins of both eggs and workers were not associated with the presence/absence of specific CC (table S1). Also, the total number of peaks extracted by group was not significantly associated with social origins (*t*-tests; eggs: *t* = 1.24, d.f. = 44, *p* = 0.22; workers: *t* = 0.61, d.f. = 44, *p* = 0.54).

## Discussion

Understanding which proximate factors influence the ability of group members to discriminate against foreigners is fundamental to gain a better understanding of the evolution and maintenance of complex social systems. Our egg-acceptance bioassays provide the first evidence in social insects that social origin of queen-laid eggs is associated with cues that interfere with nestmate discrimination. In particular, all *F. selysi* workers discriminated against foreign monogyne eggs, whereas they did not discriminate between nestmate and foreign polygyne ones. Chemical analyses confirmed that discrimination between eggs from alternative social origins could be based on quantitative differences in their chemical profiles. Finally, we showed that chemical profiles of *F. selysi* workers reflected their social origin, a result in accordance with studies in *M. barbatus* and *S. invicta* [19], [20]. Hence, this study supports the view that chemical profiles specific to each social form exist in both eggs and workers, and possibly interfere with nestmate discrimination by conspecific workers.

A somewhat surprising result of our bioassays was that both monogyne and polygyne workers rejected foreign monogyne eggs, whereas they accepted foreign polygyne ones. First, the similar behavioral response of monogyne and polygyne workers towards foreign eggs stands in contrast with the distinct behavioral response of both types of *F. selysi* workers towards foreign conspecific workers, where the level of aggression decreases with matches between social origins [38]. Second, the discrimination against foreign monogyne eggs contrasts with results in most of the Hymenopteran species studied so far, where workers tolerate eggs originating from foreign colonies, and this independently from their social origins [39]-[42], but see [43]. Altogether, these results indicate that information sources used to discriminate nestmates from non-nestmates differ when experimental intruders are eggs and workers, and reveal that eggs present (i) colony-specific cues that are used by workers to assess colony membership [22] and (ii) socially-related cues that may condition their acceptance by foreign workers.

Ultimately, the risk of egg elimination by foreign workers is unlikely to have specifically selected for the socially-related cues interfering with nestmate discrimination of eggs, as in this species, mated queens introduced in foreign colonies are generally killed before any egg production [44]. However, our artificial introduction of foreign eggs revealed that such cues exist and could be by-products of traits that are under alternative selection pressures between monogyne and polygyne colonies [28]. For instance, an egg-signal that would prevent workers from recognising maternal origin of resident eggs could have been selected in polygyne colonies to limit the costs of nepotistic behaviours [45], and by doing so, it could favor the general acceptance of polygyne eggs by foreign conspecific workers.

Proximately, the influence of social origin of eggs on their tolerance by foreign workers indicates either that (i) eggs lack reliable colony-recognition cues when they are produced in polygyne colonies, or that (ii) signals associated with polygyne origins prevent workers to perceive cues of colony-membership. The first hypothesis seems less likely, since the global-amount of extracted CC was larger in polygyne than monogyne eggs and the chemical profiles of eggs presented some differences across polygyne colonies (Mean variance in the relative amount of each peak across polygyne colonies (± SE) = 0.439±0.049). By contrast, a signal interfering with nestmate discrimination against queens has already been described in the monogyne ant *C. floridanus*, wherein workers accepted foreign conspecific queens only if they presented signals associated with high fertility [14]. Although this finding is in a different context than egg recognition, it is consistent with our second hypothesis and call for furthers studies investigating the nature of the signals (e.g. fertility signals) specific to monogyne and polygyne eggs in *F. selysi.*

Results from behavioural tests of nestmate discrimination towards eggs and workers imply that resident workers detect signals associated with the social origin of conspecific intruders in *F. selysi* (this study), [18]. Our chemical analyses revealed that monogyne and polygyne origins of eggs and workers could be discriminated on the basis of their chemical profile, a keystone in insects' communication system [3]. In social insects, divergences in chemical profiles generally result from specific genetic background or life-history traits between colonies [19], [46], [47]. In *F. selysi*, the genetic background of monogyne and polygyne colonies is unlikely to produce specific chemical signatures, as no sign of genetic differentiation or mating incompatibilities have been found between social forms in the studied populations [23], [24]. However, the number of queen per colony is associated with several life-history traits that could explain the observed differences in chemical profiles [28]. For instance, the larger volume of polygyne eggs and larger body size of monogyne workers could explain the larger global-amount of CC extracted in these two groups [27], [26]. Similarly, monogyne and polygyne colonies may suffer from different levels of queen-queen and queen-workers competitions [2], which could have selected for distinct chemical signals in their colony members. Finally, divergences between chemical profiles could be by-products of microenvironments specific to monogyne and polygyne colonies [48], [49], albeit the two types of colonies are geographically mixed in the studied population [23]. Setting up laboratory colonies wherein queen number could be manipulated might help to further disentangle whether the relative amount of CC required to influence nestmate discrimination results from the number of queen in the colony (i.e. social environment) or from the social origin of resident queens (i.e. genetic background).

Studies assessing or manipulating the availability of informations are a necessary instrument in unravelling the diversity of outcomes of social evolution and adaptive behaviour in general [50], [51]. By focusing on the social origin of intruders rather than recipient individuals, our findings shed light on the influence of social origin of eggs on their acceptance by foreign workers, and the presence of chemical compounds signalling social origins of both eggs and workers. This reveals that association between colony social structure and nestmate recognition do not necessarily reflect alternative ways for recipient workers to process colony-specific cues, but can rather result from alternative socially-influenced signals presented by conspecific intruders. The proximate and ultimate reasons for the presence of specific chemical profiles in each social form of *F. selysi* remain, however, open for further investigations.

## Supporting Information

Table S1 **Chemical compounds extracted from eggs and workers in** ***F. selysi.*** Relative amount (%) of all the chemical compounds extracted from groups of workers or eggs originating from monogyne (*Mo*) and polygyne (*Po*) colonies. Retention time (*RT*) is the mean retention time of CC across all extractions. The 15 peaks with the largest variation of relative amount between Mo and Po colonies were included in the discriminant function analyses for workers (*W*) and eggs (*E*). *P*-values remaining significant after Bonferonni corrections are indicated in **bold** (only uncorrected values are shown). Unknown compounds with identical numbers correspond to the same family of compounds. Main compounds contained in complex peaks are represented in **bold**.
(DOC)
