# Supplementary material for: Recognition in Ants: Social Origin Matters
Source: PLoS One. 2011 May 4;6(5):e19347. doi: 10.1371/journal.pone.0019347 (PMC3087756; doi:10.1371/journal.pone.0019347)
Supplement: Table S1 — Chemical compounds extracted from eggs and workers in F. selysi. Relative amount (%) of all the chemical compounds extracted from groups of workers or eggs originating from monogyne (Mo) and polygyne (Po) colonies. Retention time (RT) is the mean retention time of CC across all extractions. The 15 peaks with the largest variation of relative amount between Mo and Po colonies were included in the discriminant function analyses for workers (W) and eggs (E). P-values remaining significant after Bonferonni corrections are indicated in bold (only uncorrected values are shown). Unknown compounds with identical numbers correspond to the same family of compounds. Main compounds contained in complex peaks are represented in bold. (DOC) [file pone.0019347.s001.doc]

Table S1 - **Chemical compounds extracted from eggs and workers in *F. selysi***

|  |  |  |  |  | Workers | | |  | Eggs | | |
| --- | --- | --- | --- | --- | --- | --- | --- | --- | --- | --- | --- |
| Peaks | Name | RT | Data set | | Mo | Po | *P*-value |  | Mo | Po | *P*-value |
| 1 | Nonanal | 4.56 | W |  | **0.261** | **0.056** | **0.0004** |  | 0.076 | 0.053 | 0.0106 |
| 2 | *n*C13 | 6.42 | W |  | 0.068 | 0.043 | 0.0255 |  | - | - | - |
| 3 | *n*C14 | 7.74 |  |  | - | - | - |  | 0.066 | 0.064 | 0.5862 |
| 4 | Tridecanol | 8.23 | W |  | 0.119 | 0.078 | 0.0381 |  | - | - | - |
| 5 | *n*C15 + Unknown | 9.42 |  |  | 0.185 | 0.139 | 0.1679 |  | 0.188 | 0.170 | 0.9300 |
| 6 | *n*C16 + Unknown | 11.55 |  |  | 0.126 | 0.115 | 0.2560 |  | 0.105 | 0.102 | 0.8618 |
| 7 | Tetradecanal | 11.86 |  |  | 0.045 | 0.047 | 0.2127 |  | - | - | - |
| 8 | *n*C17 | 13.97 |  |  | 0.199 | 0.203 | 0.7940 |  | 0.108 | 0.101 | 0.4199 |
| 9 | Xi-MeC17 | 14.26 |  |  | 0.023 | 0.027 | 0.8962 |  | 0.079 | 0.061 | 0.1893 |
| 10 | Butyl dodecanoate | 15.17 |  | E | 0.035 | 0.045 | 0.0448 |  | 0.107 | 0.088 | 0.0009 |
| 11 | Xi-MeC17 | 15.39 |  |  | 0.032 | 0.033 | 0.5420 |  | 0.108 | 0.075 | 0.0519 |
| 12 | Dodecyl butyrate | 16.31 |  |  | 0.019 | 0.037 | 0.0448 |  | - | - | - |
| 13 | *n*C18 | 16.65 |  |  | 0.045 | 0.053 | 0.5566 |  | 0.210 | 0.196 | 0.1195 |
| 14 | Hexadecanal | 17.11 |  |  | 0.032 | 0.031 | 0.4721 |  | - | - | - |
| 15 | *n*C19 | 19.48 |  | E | 0.104 | 0.084 | 0.3712 |  | 0.086 | 0.073 | 0.0009 |
| 16 | Heptadecanal | 20.00 |  |  | 0.015 | 0.014 | 0.3154 |  | 0.053 | 0.044 | 0.5457 |
| 17 | Unknown 1 | 20.16 |  |  | 0.034 | 0.032 | 0.9826 |  | 0.129 | 0.084 | 0.4588 |
| 18 | Octadecanol | 20.49 |  |  | 0.123 | 0.104 | 0.0867 |  | - | - | - |
| 19 | *n*C20 (St) | 22.38 |  |  | 0.979 | 1.144 | 0.3154 |  | 6.072 | 5.335 | 0.0340 |
| 20 | Octadecanal | 22.94 |  |  | 0.072 | 0.065 | 0.8109 |  | - | - | - |
| 21 | *n*C21 | 25.21 |  |  | 0.051 | 0.049 | 0.7607 |  | 0.402 | 0.626 | 0.0223 |
| 22 | Nonadecanal | 25.87 |  | E | 0.010 | 0.008 | 0.3596 |  | 0.038 | 0.027 | 0.0016 |
| 23 | Unknown 1 | 26.28 |  |  | 0.055 | 0.080 | 0.9826 |  | 0.184 | 0.156 | 0.9387 |
| 24 | 2-Me Propyl hexadecanoate | 26.48 |  |  | 0.093 | 0.132 | 0.9307 |  | 0.144 | 0.105 | 0.0036 |
| 25 | 3-MeC21 + Nonyl undecanoate + Decyl decanoate | 27.26 |  |  | 0.038 | 0.041 | 0.0714 |  | 0.065 | 0.076 | 0.0026 |
| 26 | Unknown 1 | 27.43 |  |  | 0.041 | 0.038 | 0.7940 |  | 0.191 | 0.105 | 0.0493 |
| 27 | *n*C22 | 28.03 |  | E | 0.050 | 0.055 | 0.5276 |  | **0.318** | **0.486** | **0.0003** |
| 28 | Eicosanal | 28.63 |  |  | 0.049 | 0.040 | 0.0583 |  | - | - | - |
| 29 | 9-C23:1 | 30.05 | W | E | 1.049 | 1.572 | 0.0146 |  | **1.473** | **2.180** | **0.0003** |
| 30 | 7-C23:1 | 30.22 |  | E | 0.051 | 0.061 | 0.6013 |  | **0.084** | **0.141** | **<0.0001** |
| 31 | *n*C23 | 30.82 |  | E | 1.808 | 1.708 | 0.4199 |  | 8.884 | 12.572 | 0.0015 |
| 32 | Nonyl tridecanoate + Unknown2 | 31.12 |  |  | 0.086 | 0.072 | 0.0997 |  | 0.087 | 0.066 | 0.0042 |
| 33 | Unknown 2 | 31.27 |  |  | 0.109 | 0.124 | 0.0750 |  | 0.623 | 0.453 | 0.0018 |
| 34 | Heneicosanal | 30.77 |  | E | 0.012 | 0.009 | 0.0826 |  | 0.036 | 0.023 | 0.0009 |
| 35 | 11-,9-MeC23 | 31.74 | W |  | **0.105** | **0.506** | **<0.0001** |  | 0.858 | 1.035 | 0.0505 |
| 36 | 7-MeC23 | 31.92 | W |  | 0.059 | 0.095 | 0.0015 |  | - | - | - |
| 37 | 5-MeC23 | 32.11 | W |  | 0.062 | 0.093 | 0.0039 |  | 0.168 | 0.130 | 0.0546 |
| 38 | 2-MeC23 | 32.45 |  |  | 0.040 | 0.042 | 1.0000 |  | - | - | - |
| 39 | 3-MeC23 + Decyl dodecanoate | 32.70 | W | E | 1.024 | 1.452 | 0.0121 |  | **0.211** | **0.297** | **0.0002** |
| 40 | 5,13-diMeC23 | 32.96 |  |  | 0.136 | 0.150 | 0.4456 |  | 0.074 | 0.066 | 0.4684 |
| 41 | Unknown 1 | 33.18 |  |  | 0.029 | 0.031 | 0.9653 |  | - | - | - |
| 42 | *n*C24 | 33.44 |  |  | 0.315 | 0.303 | 0.5276 |  | 1.724 | 1.674 | 0.8605 |
| 43 | Xi-MeC24 + Decyl tridecanoate + Undecyl dodecanoate | 33.73 |  |  | 0.802 | 0.887 | 0.6476 |  | 0.091 | 0.107 | 0.1663 |
| 44 | X-Me Decyl dodecanoate | 33.99 |  |  | 0.802 | 0.871 | 0.7114 |  | - | - | - |
| 45 | X-Me Nonyl tridecanoate + X-Me Decyl dodecanoate | 34.56 |  |  | 0.449 | 0.487 | 0.9826 |  | - | - | - |
| 46 | Undecyl tridecanoate + Decyl tetradecanoate | 34.79 |  |  | 0.146 | 0.115 | 0.2293 |  | - | - | - |
| 47 | X-C25:2 + X-Me Decyl tridecanoate | 35.02 |  |  | 1.494 | 1.401 | 0.5276 |  | 0.080 | 0.049 | 0.4857 |
| 48 | 9-C25:1 + X-Me Undecyl dodecanoate | 35.45 |  |  | 4.980 | 6.167 | 0.2127 |  | 4.576 | 5.433 | 0.0134 |
| 49 | 7-C25:1 + X-Me Nonyl tetradecanoate + Xi-Me Undecyl dodecanoate | 35.58 |  |  | 0.736 | 0.807 | 0.3049 |  | 0.395 | 0.414 | 0.7170 |
| 50 | 5-C25:1 + X,X'-diMe Nonyl tridecanoate | 35.68 |  |  | 0.152 | 0.120 | 0.4857 |  | 0.036 | 0.042 | 0.4857 |
| 51 | X,X'-diMe Nonyl tridecanoate | 35.87 |  |  | 0.253 | 0.251 | 0.9134 |  | - | - | - |
| 52 | *n*C25 | 36.23 | W |  | **9.286** | **5.241** | **<0.0001** |  | 29.213 | 24.053 | 0.0130 |
| 53 | X,X'-diMe Nonyl tridecanoate | 36.38 |  |  | 0.751 | 0.687 | 0.2947 |  | 0.059 | 0.031 | 0.0381 |
| 54 | X-Me Undecyl tridecanoate | 36.59 |  |  | 0.405 | 0.384 | 0.9653 |  | - | - | - |
| 55 | X-Me Decyl tetradecanoate | 36.86 |  |  | 0.694 | 0.606 | 0.6476 |  | - | - | - |
| 56 | 13-,11-,9-MeC25 | 36.99 | W |  | 0.298 | 0.539 | 0.0092 |  | 0.959 | 0.873 | 0.1064 |
| 57 | 7-MeC25 | 37.21 |  |  | 0.275 | 0.315 | 0.6633 |  | 0.128 | 0.110 | 0.0751 |
| 58 | 5-MeC25 + X-Me Decyl tetradecanoate + X-Me Undecyl tridecanoate | 37.46 |  |  | 0.404 | 0.377 | 0.6476 |  | - | - | - |
| 59 | X-Me Decyl tetradecanoate | 37.62 |  |  | 0.139 | 0.199 | 1.0000 |  | - | - | - |
| 60 | X-Me Undecyl tetradecanoate | 37.75 |  |  | 0.048 | 0.052 | 0.8448 |  | - | - | - |
| 61 | 3-MeC25 + Decyl tetradecanoate + Dodecyl dodecanoate | 38.06 |  | E | 2.090 | 2.139 | 0.9307 |  | **0.738** | **0.930** | **0.0006** |
| 62 | 5,9-diMeC25 + X,X'-diMe Nonyl tetradecanoate | 38.23 |  |  | 0.526 | 0.625 | 0.8278 |  | 0.419 | 0.162 | 0.5566 |
| 63 | X,X'-diMe Decyl tetradecanoate | 38.44 |  |  | 0.273 | 0.255 | 0.9480 |  | - | - | - |
| 64 | *n*C26 | 38.79 |  |  | 0.285 | 0.235 | 0.2380 |  | 1.376 | 1.091 | 0.0069 |
| 65 | 3,9-+ 3,7-di-MeC25 + Decyl pentadecanoate + Undecyl tetradecanoate + Dodecyl tridecanoate | 39.01 | W | E | **0.776** | **1.276** | **<0.0001** |  | **0.285** | **0.451** | **<0.0001** |
| 66 | X-Me Decyl tetradecanoate | 39.35 |  |  | 1.283 | 1.222 | 0.5566 |  | - | - | - |
| 67 | X-Me Nonyl pentadecanoate | 40.01 |  |  | 1.809 | 1.820 | 0.7441 |  | - | - | - |
| 68 | Xi-C27:2 + X-Me Decyl pentadecanoate | 40.26 |  |  | 0.827 | 1.008 | 0.2469 |  | 0.083 | 0.108 | 0.1241 |
| 69 | Xi'-C27:2 | 40.52 |  |  | 0.272 | 0.372 | 0.1249 |  | - | - | - |
| 70 | **9-C27:1** + 7-C27:1 + X-Me Decyl tetradecanoate | 41.24 |  |  | 28.318 | 28.033 | 0.5276 |  | 13.900 | 14.655 | 0.1565 |
| 71 | 5-C27:1 + X-Me Decyl pentadecanoate | 41.51 |  |  | 2.134 | 2.049 | 0.5862 |  | 0.551 | 0.527 | 0.6367 |
| 72 | ***n*C27** + X,X'-diMe Decyl pentadecanoate | 41.80 |  |  | 3.327 | 2.515 | 0.1820 |  | 8.816 | 7.258 | 0.0305 |
| 73 | ***n*C27:2** + X,X'-diMe Decyl pentadecanoate | 42.09 |  |  | 0.914 | 0.802 | 0.5420 |  | - | - | - |
| 74 | 13-,11-,9-MeC27 | 42.59 |  |  | 0.671 | 0.645 | 0.8448 |  | 1.034 | 0.894 | 0.0211 |
| 75 | 7-MeC27 + X,X'-diMe Decyl pentadecanoate | 42.85 |  |  | 1.980 | 1.818 | 0.5134 |  | 0.258 | 0.241 | 0.5384 |
| 76 | 5-MeC27 + X-Me Undecyl pentadecanoate | 43.10 |  |  | 0.863 | 0.837 | 0.8448 |  | 0.173 | 0.188 | 0.2186 |
| 77 | 3-MeC27 + Dodecyl tetradecanoate + Decyl hextadecanoate | 43.67 | W | E | 1.301 | 2.031 | 0.0080 |  | 0.838 | 1.028 | 0.0009 |
| 78 | Unknown 1 | 43.95 |  |  | 0.553 | 0.434 | 0.2653 |  | - | - | - |
| 79 | *n*C28 + **x,y-diMeC28** | 44.68 | W | E | **0.904** | **2.308** | **<0.0001** |  | 0.755 | 0.992 | 0.0007 |
| 80 | Undecyl hextadecanoate + Dodecyl pentadecanoate + Tridecyl tetradecanoate | 44.05 |  |  | 0.307 | 0.274 | 0.9480 |  | - | - | - |
| 81 | X-Me Undecyl pentadecanoate | 44.67 |  |  | 0.313 | 0.365 | 0.7441 |  | - | - | - |
| 82 | 9,19-C29:2 | 45.90 |  |  | 2.270 | 1.984 | 0.2209 |  | 0.231 | 0.225 | 0.7170 |
| 83 | 9,21-C29:2 | 46.16 |  |  | 3.622 | 3.853 | 0.8448 |  | 0.480 | 0.549 | 0.1838 |
| 84 | 9,23-C29:2 | 46.43 |  |  | 3.097 | 3.169 | 0.7441 |  | 0.670 | 0.488 | 0.4684 |
| 85 | 9-C29:1 | 46.79 |  | E | 4.422 | 4.440 | 0.6013 |  | 4.320 | 5.161 | 0.0009 |
| 86 | 7-C29:1 | 46.89 |  |  | 2.011 | 1.907 | 0.4721 |  | 2.095 | 2.391 | 0.1305 |
| 87 | 5-C29:1 | 47.11 |  |  | 0.810 | 0.862 | 0.9653 |  | 0.438 | 0.468 | 0.2186 |
| 88 | *n*C29 + Unknown | 47.38 |  |  | 0.374 | 0.291 | 0.2469 |  | 1.830 | 1.671 | 0.2966 |
| 89 | 11-,9-MeC29 | 48.30 | W |  | 0.269 | 0.207 | 0.0240 |  | 0.355 | 0.333 | 0.4485 |
| 90 | 7-MeC29 | 48.55 |  |  | 0.090 | 0.097 | 0.6792 |  | 0.137 | 0.168 | 0.1093 |
| 91 | 9,19-C30:2 | 48.84 |  |  | 0.038 | 0.018 | 0.3712 |  | - | - | - |
| 92 | 9,21-C30:2 | 49.07 |  |  | 0.129 | 0.123 | 0.9134 |  | - | - | - |
| 93 | 3-MeC29 + Dodecyl hextadecanoate + Decyl octodecanoate | 49.33 |  |  | 0.122 | 0.145 | 0.2560 |  | 0.102 | 0.154 | 0.0040 |
| 94 | *n*C30 + Unknown 1 | 49.67 |  |  | 0.104 | 0.133 | 0.1546 |  | - | - | - |
| 95 | 12-,10-,8-MeC30 | 50.41 | W |  | **0.305** | **0.779** | **<0.0001** |  | 0.380 | 0.335 | 0.0018 |
| 96 | 9,19-C31:2 | 51.64 |  |  | 0.409 | 0.363 | 0.5134 |  | 0.036 | 0.037 | 0.3561 |
| 97 | 9,21-C31:2 | 51.89 |  |  | 2.775 | 2.687 | 0.4995 |  | 0.300 | 0.335 | 0.0634 |
| 98 | **9-C31:1** + 9,23-C31:2 | 52.25 |  | E | 0.504 | 0.509 | 0.5713 |  | **1.040** | **1.480** | **<0.0001** |
| 99 | 9,21-C33:2 | 56.65 |  |  | 0.125 | 0.126 | 0.7607 |  | - | - | - |
|  |  |  |  |  |  |  |  |  |  |  |  |
